# Supplementary material for: Hepatic-Modulatory Effects of Chicken Liver Hydrolysate-Based Supplement on Autophagy Regulation against Liver Fibrogenesis
Source: Antioxidants (Basel). 2023 Feb 15;12(2):493. doi: 10.3390/antiox12020493 (PMC9952107; doi:10.3390/antiox12020493)
Supplement: Supplementary file 1 [file antioxidants-12-00493-s001.zip › antioxidants-2172950-supplementary.pdf]

Table S1. Free amino-acid profile and imidazole-ring dipeptides in chicken-liver hydrolysate-based supplement (GBHP01^{TM}).

**Amino acid**	**Content (mg/capsule)**
L-Arginine	1.68
L-Histidine	0.37
L-Isoleucine	1.32
L-Leucine	2.60
L-Valine	1.84
L-Lysine	1.67
L-Methionine	0.55
L-Phenylalanine	1.34
L-Threonine	1.41
Tryptophan	0.31
**Total EAA**	**13.10**
**Total BCAA (leucine+isoleucine+valine)**	**5.77**
L-Alanine	1.97
β-Alanine	0.14
L-Aspartic acid	2.27
L-Cystine	N.D.
Ethanolamine	0.06
L-Glutamic acid	2.93
Glycine	1.04
L-Ornithine	0.12
o-Phosphoserine	0.72
L-Proline	1.59
L-Serine	1.54
Taurine	51.67
L-Tyrosine	0.99
**Total NEAA**	**65.04**
Anserine	0.07
Carnosine	N.D.
**Imidazole-ring dipeptide**	**0.07**

N.D.: not detected.
